# Malin restoration as proof of concept for gene therapy for Lafora disease

**DOI:** 10.1093/braincomms/fcac168

**Published:** 2022-06-23

**Authors:** Olga Varea, Joan J Guinovart, Jordi Duran

**Affiliations:** Institute for Research in Biomedicine (IRB Barcelona), The Barcelona Institute of Science and Technology, Barcelona 08028, Spain; Institute for Research in Biomedicine (IRB Barcelona), The Barcelona Institute of Science and Technology, Barcelona 08028, Spain; Centro de Investigación Biomédica en Red de Diabetes y Enfermedades Metabólicas Asociadas (CIBERDEM), Madrid 28029, Spain; Department of Biochemistry and Molecular Biomedicine, University of Barcelona, Barcelona 08028, Spain; Institute for Research in Biomedicine (IRB Barcelona), The Barcelona Institute of Science and Technology, Barcelona 08028, Spain; Centro de Investigación Biomédica en Red de Diabetes y Enfermedades Metabólicas Asociadas (CIBERDEM), Madrid 28029, Spain; Institut Químic de Sarrià (IQS), Universitat Ramon Llull (URL), Barcelona 08017, Spain; Institute for Bioengineering of Catalonia (IBEC), The Barcelona Institute of Science and Technology, Barcelona 08028, Spain

**Keywords:** glycogen, Lafora disease, neurodegeneration, neuroinflammation, gene therapy

## Abstract

Lafora disease is a fatal neurodegenerative childhood dementia caused by loss-of-function mutations in either the laforin or malin gene. The hallmark of the disease is the accumulation of abnormal glycogen aggregates known as Lafora bodies (LBs) in the brain and other tissues. These aggregates are responsible for the pathological features of the disease. As a monogenic disorder, Lafora disease is a good candidate for gene therapy-based approaches. However, most patients are diagnosed after the appearance of the first symptoms and thus when LBs are already present in the brain. In this context, it was not clear whether the restoration of a normal copy of the defective gene (either laforin or malin) would prove effective. Here we evaluated the effect of restoring malin in a malin-deficient mouse model of Lafora disease as a proof of concept for gene replacement therapy. To this end, we generated a malin-deficient mouse in which malin expression can be induced at a certain time. Our results reveal that malin restoration at an advanced stage of the disease arrests the accumulation of LBs in brain and muscle, induces the degradation of laforin and glycogen synthase bound to the aggregates, and ameliorates neuroinflammation. These results identify malin restoration as the first therapeutic strategy to show effectiveness when applied at advanced stages of Lafora disease.

## Teaser

Lafora disease is a fatal childhood dementia caused by mutations in either laforin or malin genes. Duran *et al*. evaluated malin restoration as a proof of concept for a therapeutic approach for malin-deficient Lafora disease. They show that malin restoration is an effective treatment even when applied at an advanced stage of the disease.

## Graphical Abstract

**Graphical Abstract:**
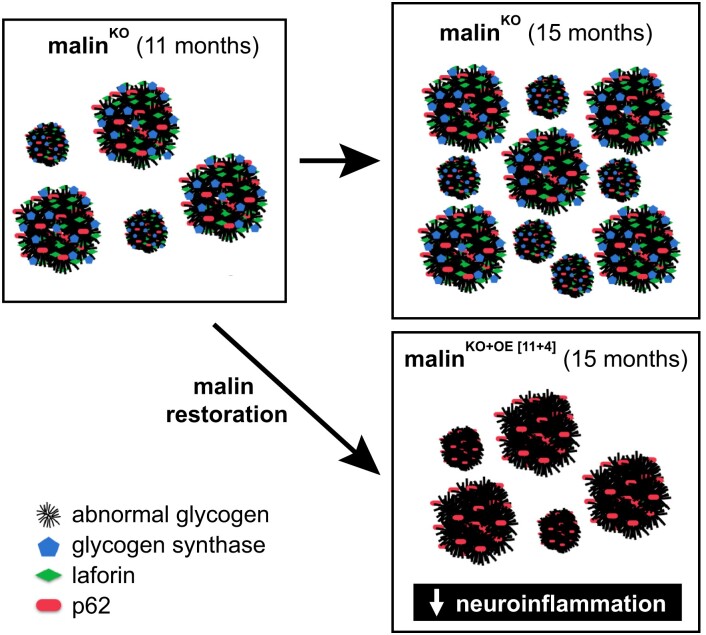

## Introduction

Lafora disease (EPM2, OMIM254780) is a fatal neurodegenerative childhood dementia that typically manifests with epilepsy during adolescence. It is caused by disruptive mutations in either the laforin (*EPM2A*) or malin (*EPM2B*) gene.^1–4^ Laforin is a dual-specificity phosphatase with the ability to bind directly to glycogen through a carbohydrate-binding domain.^5–7^ Malin is an E3-ubiquitin ligase that binds laforin and promotes the degradation of proteins involved in glycogen metabolism, including laforin and muscle glycogen synthase (MGS).^2,3,8,9^ Mutations in either of the two genes cause an undistinguishable phenotype in patients, as well as in mouse models of the disease. Current treatments are only palliative, oriented to control the frequency and severity of the epileptic seizures.

During the progression of Lafora disease, several tissues, including the brain and skeletal muscle, accumulate large aggregates of aberrant glycogen. These aggregates, known as Lafora bodies (LBs), also contain a variety of proteins, including MGS, laforin (in malin-deficient conditions), and p62,^10–12^ an autophagy adaptor that participates in the aggregation of abnormal glycogen into LBs.^13^ In the brain, LBs are found in neurons (neuronal LBs, nLBs), and in astrocytes [‘corpora amylacea like’ (CAL) because of their resemblance to corpora amylacea—glycogen aggregates that accumulate in the brain with age^12^]. The two types of LBs differ not only in the cell type in which they are located but also in their intracellular distribution, morphology, and contribution to the pathology.^12–14^

Several groups, including us, have definitively demonstrated that the accumulation of LBs underlies the neuroinflammation and susceptibility to epilepsy characteristic of Lafora disease.^10,11,14–20^ Therefore, the strategies currently being investigated as potential therapeutic approaches for Lafora disease focus on promoting the degradation of LBs^17^ or arresting their formation.^11,18,21,22^ The suppression of MGS arrests LB accumulation in malin- and laforin-deficient mice but it is only effective in stopping neuroinflammation when applied at early stages of the disease.^11,18,21,22^ This observation thus highlights the need for alternative approaches that offer more effective treatment of the disease, particularly at advanced stages.

As an autosomal recessive inherited single-gene disorder (caused by mutations in either laforin or malin), Lafora disease is a good candidate for a gene therapy-based approach. Most patients are diagnosed with the first symptoms of the disease and thus when LBs have already accumulated in the brain. It remained unclear whether gene therapy would provide an effective treatment at this stage, since the mechanisms linking the lack of malin or laforin with the accumulation of LBs are still unclear. In this work, we evaluated malin restoration as a proof of concept for a therapeutic approach for malin-deficient Lafora disease. To this end, we generated a malin-deficient mouse (malin^KO^) in which malin expression can be initiated at a certain time (malin^KO+OE^). Our results show that malin expression arrests the accumulation of LBs and promotes a reduction of the inflammatory response even when induced at an advanced stage. Interestingly, malin restoration also triggers the degradation of MGS and laforin bound to LBs. This is the first time that a therapeutic intervention has proven effective when applied at an advanced stage of the disease. Our results provide a proof of concept for the development of a malin gene therapy-based approach for Lafora disease.

## Materials and methods

### Animals

All experimental protocols were approved by the Barcelona Science Park Animal Experimentation Committee and were carried out following Spanish (BOE 34/11370-421, 2013) and European Union (2010/63/EU) regulations, and The National Institutes of Health guidelines for the care and use of laboratory animals. Experiments were conducted using littermates, and males and females were included in each group. Where necessary, groups included multiple litters for statistical power. The experiments were carried out in C57/Bl6 mice aged between 4 and 15 months. The design of specific genotyping probes and genotyping was performed by TransnetYX^R^.

To generate mice with inducible malin expression (malin^OE^), an expression cassette composed of the CMV early enhancer/chicken β-actin (CAG) promoter, a floxed 2xSTOP cassette and the mouse malin cDNA was introduced into the Col1A1 locus by homologous recombination in G4 (129/B6/F1) mouse embryonic stem (ES) cells. The targeted ES cell clones were identified by long-range PCR and confirmed by Southern blot analysis. The targeted ES cell clones were then injected into C57BL/6J mouse blastocysts and implanted into pseudo-pregnant females. Male chimeras were selected for breeding with wild-type C57BL/6J females to generate the transgenic line. Germ-line transmission of the transgene was confirmed by PCR and Southern blot. Transgenic mice were then mated with Cre-ERT2 Cre transgenic mice {B6.Cg-Ndor1Tg[UBC-cre/oestrogen receptor-ligand binding Domain 2 (ERT2)]1Ejb/2J, Jackson mice}, which ubiquitously express a tamoxifen-inducible Cre-recombinase. We then mated the resulting mice with malin^KO^ mice^23^ to generate malin^KO+OE^ animals. Genotyping of the alleles involved was performed using specific probes designed and applied by TransnetYX®.

### Tamoxifen administration

Tamoxifen was administered by intraperitoneal injections. Three doses of 0.225 mg tamoxifen/g of body weight were injected into each mouse on alternative days. After tamoxifen administration, the stop signal that precedes the malin transgene is permanently removed, allowing the expression of malin for the rest of the experiment. Tamoxifen solution was prepared at 20 mg/ml, first dissolved in 100% EtOH and completed with filtered Sunflower seed oil (Sigma). Finally, tamoxifen was left overnight with agitation at 37°C and kept at 4°C for its immediate use.

### Histology

Animals were first deeply anesthetized with thiobarbital (Braun) for transcardiac perfusion with 4%paraformaldehyde in phosphate buffer saline. Tissues from brain and skeletal muscle (quadriceps) were collected and embedded in paraffin. Sections with a thickness of 3 µm were obtained for each sample using a microtome (Leica). For periodic acid-Schiff staining (PAS) and immunofluorescence, we followed the previous protocols described.^11^ The following primary antibodies were used: anti-MGS (3886, Cell signaling); anti-laforin (mouse monoclonal, clone Ab2, gift from Dr Santiago Rodriguez de Córdoba); anti-GFAP (MAB360, Merck Millipore); anti-p62 (Progen, GP62-C); and anti-WGA (wheat germ agglutinin)-AlexaFluor^TM^ 488 conjugated (Thermofisher, W11261). The correspondent secondary antibodies used were as follows: anti-rabbit DyLight 594 (DI-1094, Vector); anti-rabbit Alexa Fluor 488 (A11034, ThermoFisher); anti-mouse Alexa Fluor 568 (A11031, Invitrogen); or/and anti-mouse IgG Alexa Fluor 488 (405319, Biolegend); and anti-guinea pig Cy3, in combination with DAPI (Sigma). For brightfield labelling, the anti-CD11b antibody (ab133357, Abcam) and the secondary antibody BrightVision poly HRP-Anti-rabbit IgG (DPVR-110HRP, ImmunoLogic) were used. Serial brain sections containing the CA1/CA2/CA3 and the dentate gyrus were analysed for consistency as previously described.^11^ In this regard, 3–4 non-consecutive sections from each sample were analysed. The number of mice is indicated in each figure. NDPview 2 software (Hamamatsu, Photonics, France) with a gamma correction set at 1 for fluorescence was used to analyse the images. Brightness was adjusted to improve printing quality to 130% in each colour channel and image. The stainings were analysed by the digital software QuPath.^24^ For nLB detection, particle identification was established at a 2–200 µm^2^ background radius and 0.8–1 circularity. CAL was defined as the subtraction of the number of nLBs from the total number of particles detected per region. A ‘Cell detection’ tool was used to identify aggregates. Plot profiles were generated in FIJI software by drawing a line crossing the geometrical centre of the LB and using the PlotProfile tool. To detect each p62-positive aggregate, the acquisition settings were fixed in saturating conditions for one channel to determine the fluorescence intensity of the rest of the channels. The ‘% Positive pixel’ tool was used to calculate percentage of positive pixel of the total hippocampal area (region of interest, ROI) in each section for GFAP and CD11b stainings in brain, and MGS and p62 in muscle. The ‘% of double positives (MGS/p62 or laforin/p62)’ was determined by establishing a threshold of intensity for the negative laforin or MGS staining (fixed as *I*_max_ ≤ 10) in the ROI (CA2/CA3 region for the hippocampus, Hp, prefrontal cortical area, Cx, or central region of quadriceps muscle sections). Intensities of laforin, MGS, and p62 staining were measured using QuPath software in LB detected as described above in the same ROIs.

### Glycogen quantification

Before decapitation, mice were deeply anesthetized with thiobarbital (Braun). Brain and quadriceps muscles were removed, frozen, and pulverized in liquid nitrogen and stored at −80°C. For glycogen measurement, tissues were boiled in 30% KOH for 15 min and glycogen was precipitated in 66% EtOH. The total amount of glycogen was determined using an amyloglucosidase-based assay, as described previously.^11,25^

### Western blot

Frozen brain and quadriceps muscles were homogenized in 25 mM Tris–HCl (pH 7.4), 25 mM NaCl, 1% Triton X-100, 0.1% sodium dodecyl sulfate (SDS), 0.5 mM ethylene glycol tetraacetic acid (EGTA), 10 mM sodium pyrophosphate, 1 mM sodium orthovanadate, 10 mM NaF, 25 nM okadaic acid, and a protease inhibitor cocktail tablet (Roche). Soluble and insoluble fractions were obtained as previously described.^23^ Briefly, total homogenates were centrifuged at 13,000 *g* for 15 min at 4°C. The pellet containing the insoluble fraction was recovered in the same volume as the supernatant corresponding to the soluble fraction. Samples were loaded on 10% acrylamide gels for SDS-PAGE and transferred to Immobilon membranes (Millipore). The following primary antibodies were used: anti-GS (rabbit, 3886, Cell Signalling); anti-laforin (mouse, 3.5.5, kindly provided by Dr Santiago Rodríguez de Córdoba); and anti-p62 (guinea pig, Progen). The following secondary antibodies were used: anti-rabbit and anti-mouse IgG-HRP (GE Healthcare); and anti-guinea pig HRP (Jackson Immuno Research). Proteins were detected by the ECL method (Immobilon Western Chemiluminescent HRP Substrate, Millipore), and loading control of the western blot membrane was performed using the REVERT (LI-COR Bioscience) total protein stain.

### Quantitative (q)PCR

Frozen brains and quadriceps muscles were used to obtain total RNA using Trizol reagent (LifeTechnologies, Carlsbad, CA, USA). Purification was performed with RNeasy Mini Kit (Qiagen, Hilden, Germany) and RNAs were then treated with DNase I (Qiagen). cDNA was obtained using the qScript cDNA Synthesis Kit (Quanta Biosciences, Beverly, MA, USA). A Quantstudio 6 Flex instrument was used (Applied Biosystems, Foster City, CA, USA) for the qPCR reaction The following mouse-specific SYBRgreen set of primers (Sigma, Madrid, Spain) was used: *18S* rRNA housekeeping gene (forward: ATTAAGTCCCTGCCCTTTGTACAC, reverse: TAGATAGTCAAGTTCGACCGTCTTCTC), *IL-6* (forward: TAGTCCTTCCTACCCCAATTTCC, reverse: TTGGTCCTTAGCCACTCCTTC), *CXCL10* (forward: CCGTCATTTTCTGCCTCATC, reverse: CTCGCAGGGATGATTTCAAG), malin (forward: TCACCAACGACTGCCATGTG, reverse: TTCCAGCAGGTGCAAAGTCC), GAPDH (forward: TGAAGCAGGCATCTGAGGG, reverse: CGAAGGTGGAAGAGTGGGAG). For representation of the results, relative expression (2^-ΔΔCt^) was calculated with respect to control mice.

### Statistics

Significance between two variables was analysed using the Student’s *t*-test performed with the GraphPad Prism software (La Jolla, CA, USA). One-way ANOVA with Tukey *post hoc* analysis was used for comparison of the groups using multiple comparisons in GraphPad Prism software. Alternatively, the Student’s *t*-test was applied when required. The following *P*-values were considered statistically significant: *P*-*value* ≤ *0.05*(*), *P-value < 0.01* (**), and *P-value < 0.001* (***).

### Data availability

Raw data and images are available upon request from the corresponding author.

## Results

### Generation of malin^KO+OE^ mice

To evaluate the impact of restoring malin in a mouse model of Lafora disease lacking malin (malin^KO^), we generated mice containing an inducible malin expression cassette (malin^OE^) (see Materials and methods and Supplementary Fig. 1). These animals were then combined with UBC-Cre-ERT2 mice, in which a tamoxifen-inducible Cre-recombinase is ubiquitously expressed. The resulting mice were then crossed with malin^KO^ mice to obtain the malin^KO^ model in which malin expression can be permanently restored at a certain time (malin^KO+OE^). See Supplementary Fig. 1 for a scheme of the experimental planning. Malin expression was induced by intraperitoneal tamoxifen injection at the time-points of interest (see Materials and methods). qPCR revealed that brain malin expression in induced malin^KO+OE^ ranged from levels comparable to those of control mice to 10-fold higher (Supplementary Fig. 1). In skeletal muscle, malin expression was up to 100-fold that of control mice (Supplementary Fig. 1). This observation thus indicates that activation of gene expression was more effective in this tissue than in the brain.

### Malin restoration at an early stage of the disease affects LB composition

**Figure 1:**
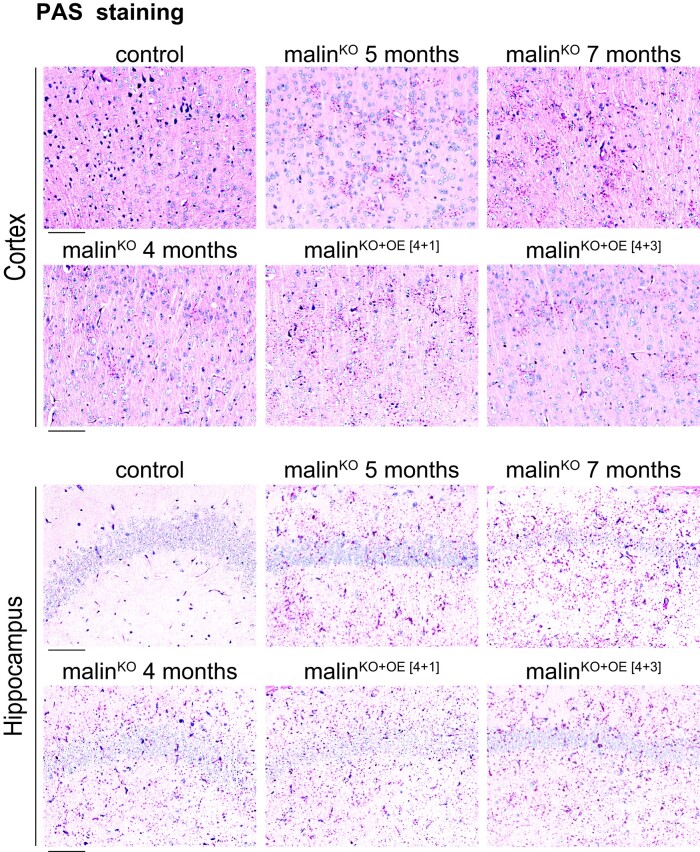
**Malin restoration in young malin^KO^ mice. PAS staining in brain.** Representative images from control, 4-, 5-, and 7-month-old malin^KO^ and malin^KO+OE^ mice after 1 or 3 months of malin restoration. The upper panels show a cortical region and the lower panels the CA2/CA3 hippocampal region. Scale bar: 100 µm. *n = 4*–*5 mice*.

To study the effects of malin reintroduction at an early stage of Lafora disease, malin expression was induced in 4-month-old malin^KO+OE^ mice, an age at which malin^KO^ mice already accumulate abundant LBs in the brain.^11,14^ These animals were analysed 1 month and 3 months after the induction, referred to as malin^KO+OE[4+1]^ and malin^KO+OE[4+3]^, respectively (Supplementary Fig. 1). Brain LBs were first examined by PAS, which detects glycogen. Malin^KO+OE[4+1]^ and malin^KO+OE[4+3]^ mice showed a comparable presence of LBs to that of 4-month-old malin^KO^ brains (Fig. 1), indicating that malin expression did not induce the degradation of pre-existing glycogen aggregates.

**Figure 2:**
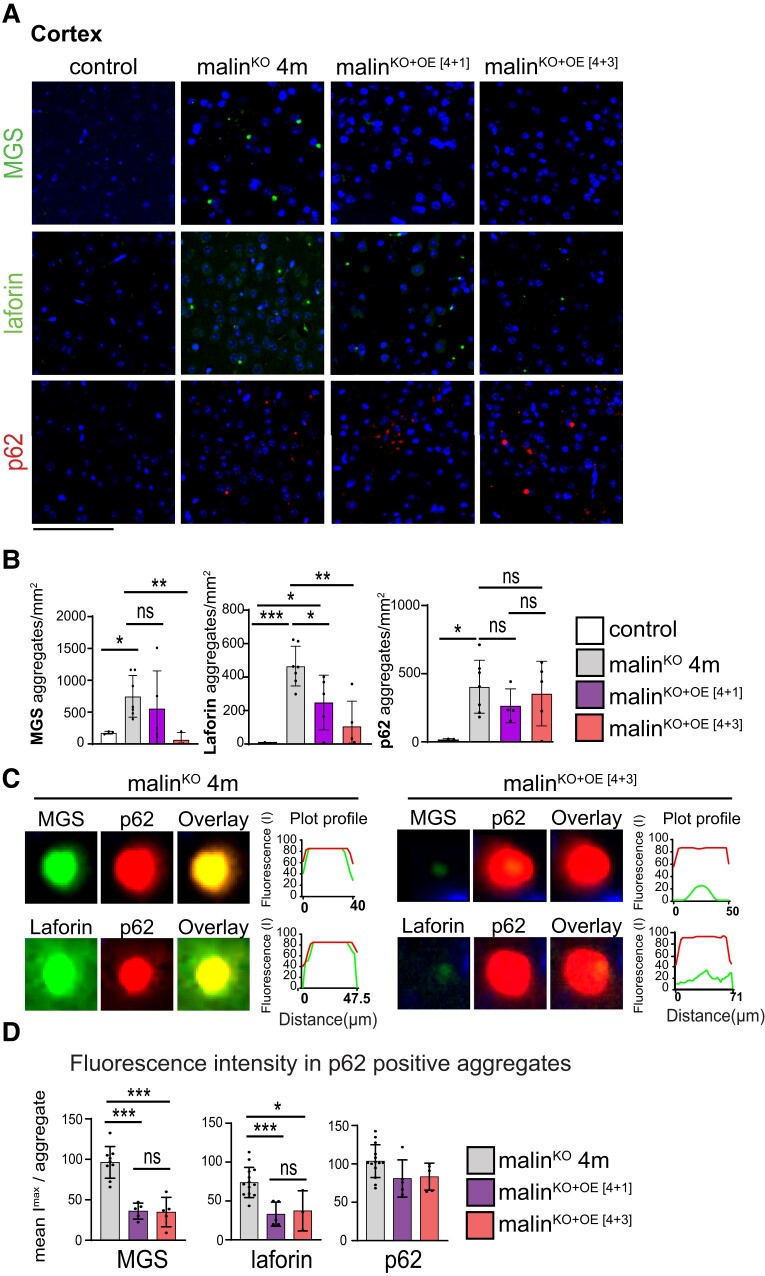
**LB component analysis of young malin^KO^ mice after malin restoration.** (**A**) Representative immunofluorescence images from the cortical region obtained with anti-MGS (green), anti-laforin (green), or anti-p62 (red) stainings in combination with DAPI (blue). Control and 4-month-old malin^KO^ (malin^KO^ 4 m), malin^KO+OE[4+1]^, and malin^KO+OE[4+3]^ mice are shown. Scale bar: 100 µm. (**B**) Quantification of number of aggregates per area (mm^2^) detected by MGS, laforin, or p62 staining. ANOVA with Tukey’s multiple comparison test and unpaired *t*-test. MGS: control versus malin^KO^ 4 months: *P* = 0.01, malin^KO^ 4 months versus malin^KO+OE[4+1]^: *P* = 0.48, malin^KO^ 4 months versus malin^KO+OE[4+3]^: *P* = 0.0092, malin^KO+OE[4+1]^ versus malin^KO+OE[4+3]^: *P* = 0.21. Laforin: control versus malin^KO^ 4 months: *P* = 0.0001, control versus malin^KO+OE[4+1]^: *P* = 0.021, malin^KO^ 4 months versus malin^KO+OE[4+1]^: *P* = 0.022, malin^KO^ 4 months versus malin^KO+OE[4+3]^: *P* = 0.009. p62: control versus malin^KO^ 4 months: *P* = 0.01, malin^KO^ 4 months versus malin^KO+OE[4+1]^: *P* = 0.23, malin^KO^ 4 months versus malin^KO+OE[4+3]^: *P* = 0.69, malin^KO+OE[4+1]^ versus malin^KO+OE[4+3]^: *P* = 0.51. *P-value ≤ 0.05*(*), *P-value < 0.01* (**), and *P-value < 0.001* (***), unpaired *t*-test. (**C**) Magnification of representative LBs found in 4-month-old malin^KO^ mice (left) compared with malin^KO+OE[4+3]^ mice (right). MGS and laforin (green) were co-stained with p62 (red), the overlay image for each one is shown (merge). Fluorescent profile along a line across the LB through its geometrical centre is shown in each case. The size of the fluorescent particle is indicated in each graphic. (**D**) Fluorescent intensity of MGS and laforin in p62-positive LBs from malin^KO^, malin^KO+OE[4+1]^, and malin^KO+OE[4+3]^ mice. ANOVA with Tukey’s multiple comparison test and unpaired *t*-test. MGS, malin^KO^ 4 months versus malin^KO+OE[4+1]^: *P ≤* 0.0001, malin^KO^ 4 months versus malin^KO+OE[4+3]^: *P ≤* 0.0001, malin^KO+OE[4+1]^ versus malin^KO+OE[4+3]^: *P* = 0.9. Laforin: malin^KO^ 4 months versus malin^KO+OE[4+1]^: *P* = 0.0006, malin^KO^ 4 months versus malin^KO+OE[4+3]^: *P* = 0.013, malin^KO+OE[4+1]^ versus malin^KO+OE[4+3]^: *P* = 0.77. p62: malin^KO^ 4 months versus malin^KO+OE[4+1]^: *P* = 0.065, malin^KO^ 4 months versus malin^KO+OE[4+3]^: *P* = 0.075, malin^KO+OE[4+1]^ versus malin^KO+OE[4+3]^: *P* = 0.86. *P-value ≤ 0.05*(*), *P-value < 0.01* (**), and *P-value < 0.001* (***). Data are shown as mean ± SD. Each dot represents one mouse (*n = 4*–*8* as indicated).

To further study LB composition, we next performed fluorescent immunostainings in serial brain sections using antibodies against LB-bound proteins, namely MGS, laforin, and p62. MGS and laforin progressively accumulate bound to aberrant glycogen during the course of the disease.^11^ Interestingly, the cortex of the malin^KO+OE^ groups showed a reduced number of laforin- and MGS-positive aggregates (Fig. 2A and B). In fact, malin^KO+OE[4+3]^ animals showed a virtual absence of MGS- and laforin-positive aggregates. In contrast, p62-positive aggregates were maintained in malin^KO+OE[4+1]^ and malin^KO+OE[4+3]^ mice (Fig. 2A and B). Therefore, while in malin^KO^ brains the vast majority of p62-positive aggregates co-localized with MGS and laforin, as previously described,^12^ in malin^KO+OE^ animals there was a considerable number of p62-positive aggregates lacking MGS and laforin staining. To visualize this effect, representative aggregates double stained for MGS/p62 and laforin/p62 are shown (Fig. 2C). A line fluorescence profile across the geometrical centre of the LBs shows the relative presence of these proteins, indicating that in malin^KO+OE[4+3]^ a significant population of p62 aggregates do not contain MGS or laforin.

To quantify this effect, we measured the mean fluorescence intensity of laforin and MGS in p62-positive aggregates in the cortex (Fig. 2C) and hippocampus (Supplementary Fig. 2) of malin^KO^ and malin^KO+OE^ mice. Malin^KO+OE[4+1]^ and malin^KO+OE[4+3]^ mice showed a reduction in the fluorescence intensity of both laforin and MGS at the p62-positive aggregates compared with age-matched malin^KO^ mice. The mean intensity of p62 fluorescence, which was measured as a positive control, remained invariable between the three groups (Fig. 2D). Similar results were obtained when analysing the hippocampal region (Supplementary Fig. 2). All together, malin reduced the presence of MGS and laforin, but not p62, in the LBs.

### Malin expression arrests brain LB accumulation at an advanced stage of Lafora disease

**Figure 3:**
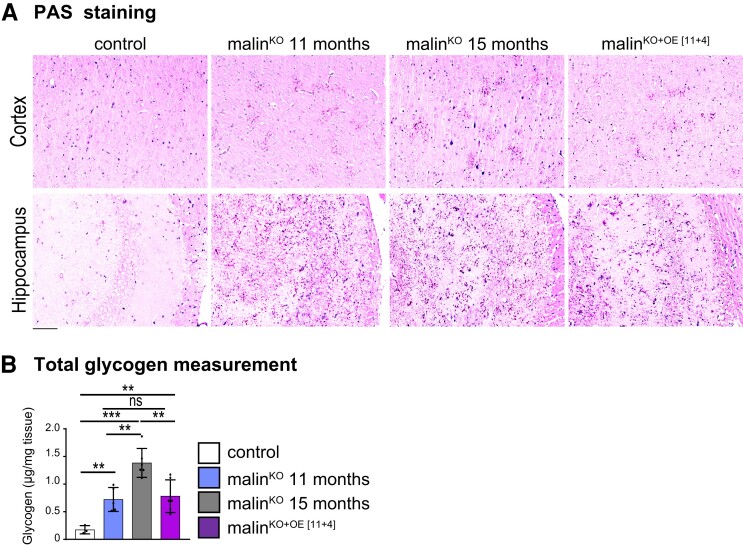
**Malin restoration at an advanced stage of Lafora disease in malin^KO^ mice.** (**A**) PAS staining in brain. Representative images of cortical and CA2/CA3 hippocampal region from control, 11-month-old malin^KO^, 15-month-old malin^KO^, and malin^KO+OE[11+4]^ mice are shown. ANOVA with Tukey’s multiple comparison test and unpaired *t*-test. Control versus malin^KO^ 11 months: *P* = 0.00003, control versus malin^KO^ 15 months: *P* = 0.0001, control versus malin^KO+OE[11+4]^: *P* = 0.0094, malin^KO^ 11 months versus malin^KO^ 15 months: *P* = 0.0032, malin^KO^ 11 months versus malin^KO+OE[11+4]^: *P* = 0.96, malin^KO^ 15 months versus malin^KO+OE[11+4]^: *P* = 0.0094. Scale bar: 100 µm. (**B**) Total brain glycogen was determined from the same groups (µg/mg of tissue). Each dot represents one mouse (*n = 3*–*6*).

To study the effectiveness of malin restoration in a scenario more representative of a patient with clinical symptoms of Lafora disease, we next induced malin expression in 11-month-old malin^KO+OE^ mice, a time-point at which malin^KO^ mice present a high abundance of LBs and a strong inflammatory response, as reflected by profound astrocytosis and microgliosis.^10,11,26^ Mice were analysed 4 months after the induction of malin expression (referred to as malin^KO+OE[11+4]^). PAS staining showed that LBs were more abundant in 15-month-old than in 11-month-old malin^KO^ animals (Fig. 3A). Interestingly, malin^KO+OE[11+4]^ mice presented fewer glycogen aggregates than age-matched 15-month-old malin^KO^ counterparts, and comparable to 11-month-old malin^KO^ animals. Total brain glycogen corresponds with the number of LBs.^10,11,18,21^ Accordingly, malin^KO+OE[11+4]^ mice showed a significantly lower glycogen level than 15-month-old malin^KO^ mice and a comparable level to that of 11-month-old malin^KO^ mice. These results indicate that, although malin expression did not result in the elimination of the pre-existing glycogen, it did arrest its further accumulation (Fig. 3B).

### Malin expression reduces brain insoluble laforin and MGS at an advanced stage of Lafora disease

**Figure 4:**
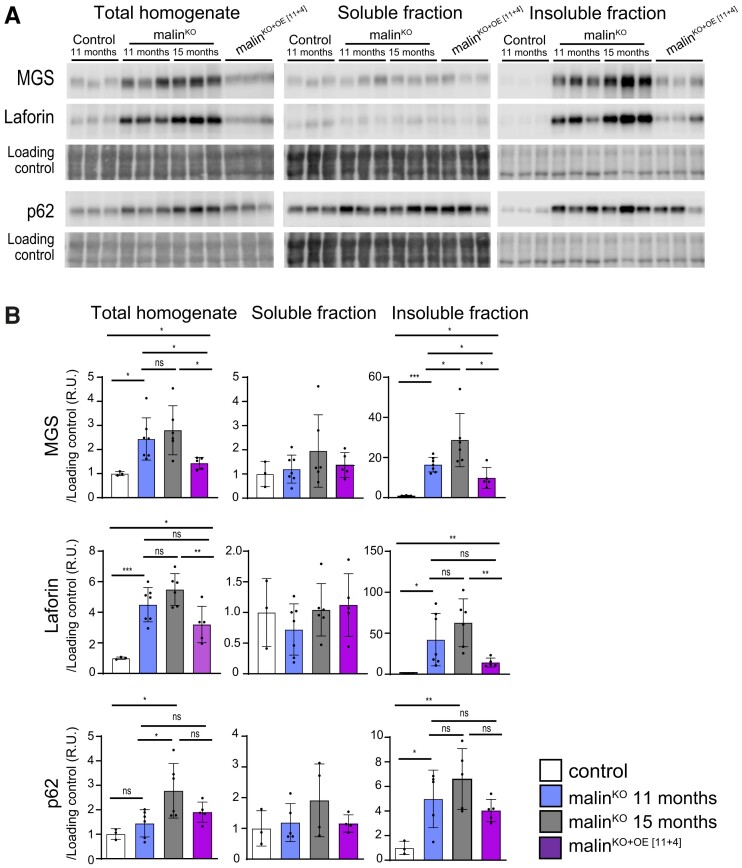
**Soluble and insoluble brain fractions analysis.** (**A**) Representative western blots of total brain homogenate, soluble, and insoluble fractions are shown for MGS, laforin, and p62. The images shown are cropped to show the band of interest, for full images see Supplementary material. Three samples from the same group were run for these images from a total of 3–6 mice per group. Loading control: LICOR—revert staining. (**B**) Quantifications of protein detection by western blot. Relative optical density units were related to loading control and normalized respect to control mice. Data are shown as mean ± SD. ANOVA with Tukey’s multiple comparison test and unpaired *t*-test. MGS total homogenate: control versus malin^KO^ 11 months: *P* = 0.025, control versus malin^KO^ 15 months: *P* = 0.02, malin^KO^ 15 months versus malin^KO[11+4]^: *P* = 0.017, malin^KO^ 11 months versus malin^KO+OE[11+4]^: *P* = 0.033, control versus malin^KO+OE[11+4]^: *P* = 0.028. MGS insoluble fraction: control versus malin^KO^ 11 months: *P* = 0.01, control versus malin^KO^ 15 months: *P* = 0.0001, malin^KO^ 15 months versus malin^KO+OE[11+4]^: *P* = 0.015, malin^KO^ 11 months versus malin^KO+OE[11+4]^: *P* = 0.026, control versus malin^KO+OE[11+4]^: *P* = 0.029. Laforin total homogenate: control versus malin^KO^ 11 months: *P* = 0.0008, control versus malin^KO^ 15 months: *P* = 0.02, malin^KO^ 15months versus malin^KO+OE[11+4]^: *P* = 0.0076, malin^KO^ 11 months versus malin^KO+OE[11+4]^: *P* = 0.08, control versus malin^KO+OE[11+4]^: *P* = 0.021. Laforin insoluble fraction: control versus malin^KO^ 11 months: *P* = 0.04, control versus malin^KO^ 15 months: *P* = 0.0094, malin^KO^ 15 months versus malin^KO+OE[11+4]^: *P* = 0.0054, malin^KO^ 11 months versus malin^KO+OE[11+4]^: *P* = 0.085, control versus malin^KO+OE[11+4]^: *P* = 0.0062. p62 total homogenate fraction: control versus malin^KO^ 11 months: *P* = 0.105, control versus malin^KO^ 15 months: *P* = 0.032, malin^KO^ 11 months versus malin^KO+OE[11+4]^: *P* = 0.54, malin^KO^ 15 months versus malin^KO+OE[11+4]^: *P* = 0.0511. p62 insoluble fraction: control versus malin^KO^ 11 months: *P* = 0.04, control versus malin^KO^ 15 months: *P* = 0.010, malin^KO^ 11 months versus malin^KO^ 15 months: *P* = 0.24, malin^KO^ 11 months versus malin^KO+OE[11+4]^: *P* = 0.67, malin^KO^ 15 months versus malin^KO+OE[11+4]^: *P* = 0.14. *P-value ≤ 0.05*(*), *P-value < 0.01* (**), and *P-value < 0.001* (***). Each dot represents one mouse (*n = 3*–*6*).

**Figure 5:**
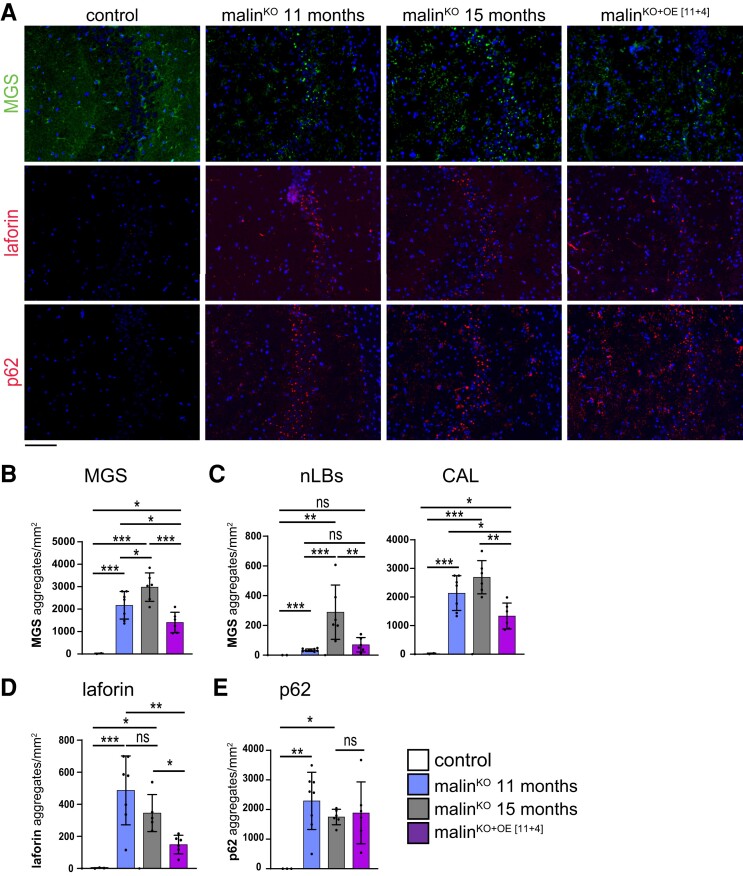
**MGS, laforin, and p62 analysis in the hippocampus of malin^KO^ mice after malin restoration at an advanced stage of Lafora disease.** (**A**) Immunofluorescence images from the hippocampal region using anti-MGS (green), anti-laforin (red), or anti-p62 (red) antibodies combined with DAPI staining in each group. (**B**) Quantification of number of particles per area (mm^2^) in CA2/CA3 region stained with anti-MGS antibody. ANOVA with Tukey’s multiple comparison test. MGS total aggregates: control versus malin^KO^ 11 months: *P* = 0.00038, control versus malin^KO^ 15 months: *P* = 0.0008, control versus malin^KO+OE[11+4]^: *P* = 0.064, malin^KO^ 11 months versus malin^KO^ 15 months: *P* = 0.0697, malin^KO^ 11 months versus malin^KO+OE[11+4]^: *P* = 0.0287, malin^KO^ 15 months versus malin^KO+OE[11+4]^: *P* = 0.0006. nLBs: control versus malin^KO^ 11 months: *P* = 0.0006, control versus malin^KO^ 15 months: *P* = 0.0078, control versus malin^KO+OE[11+4]^: *P* = 0.1, malin^KO^ 11 months versus malin^KO^ 15 months: *P* = 0.0009, malin^KO^ 11 months versus malin^KO+OE[11+4]^: *P* = 0.06, malin^KO^ 15 months versus malin^KO+OE[11+4]^: *P* = 0.0175. CAL, control versus malin^KO^ 11 months: *P* = 0.0023, control versus malin^KO^ 15 months: *P* = 0.0008, control versus malin^KO+OE[11+4]^: *P* = 0.081, malin^KO^ 11 months versus malin^KO^ 15 months: *P* = 0.12, malin^KO^ 11 months versus malin^KO+OE[11+4]^: *P* = 0.02, malin^KO^ 15 months versus malin^KO+OE[11+4]^: *P* = 0.0011. (**C**) Quantification of nLBs and CALs from the hippocampus in each group. Quantification of number of particles per area (mm^2^) in CA2/CA3 hippocampal region stained with laforin (**D**) or p62 (**E**). ANOVA with Tukey’s multiple comparison test. Scale bar: 100 µm. In all graphics, data are shown as mean ± SD. *P-value ≤ 0.05*(*), *P-value < 0.01* (**), and *P-value < 0.001* (***). Each dot represents one mouse (*n = 3*–*6* as indicated in the graphic).

To further assess the effects of malin expression, we measured the levels of LB-bound proteins, namely MGS, laforin, and p62, using biochemical and imaging techniques. We first analysed total brain homogenates by western blot, as well as the soluble and insoluble fractions, the latter enriched in LB-associated proteins.^23^ Malin^KO+OE[11+4]^ mice showed significantly reduced levels of insoluble laforin and MGS compared with age-matched, 15-month-old malin^KO^ mice, the insoluble levels even being below the levels found in 11-month-old malin^KO^ mice. However, the levels of p62 in malin^KO+OE[11+4]^ mice were comparable to those of age-matched malin^KO^ mice (Fig. 4A and B). These results again indicate that malin induced the degradation of some components of pre-existing LBs, such as laforin and MGS, but not p62. We next performed immunofluorescence stainings for MGS, laforin, and p62 in both hippocampus (Fig. 5A) and cortex (Supplementary Fig. 3). Quantifications of these images showed that the total number of MGS-positive aggregates in the hippocampal and cortical regions of malin^KO+OE[11+4]^ mice was greatly reduced when compared with age-matched malin^KO^ mice, reaching levels below those of 11-month-old malin^KO^ mice (Fig. 5B and Supplementary Fig. 3, respectively). This reduction affected both the number of MGS-positive-nLBs and MGS-positive-CAL (Fig. 5C). Similar results were obtained with the quantification of laforin-positive aggregates in both regions (Fig. 5D and Supplementary Fig. 3). In contrast, the number of p62 aggregates was not decreased in malin^KO+OE[11+4]^ mice (Fig. 5E and Supplementary Fig. 3).

**Figure 6:**
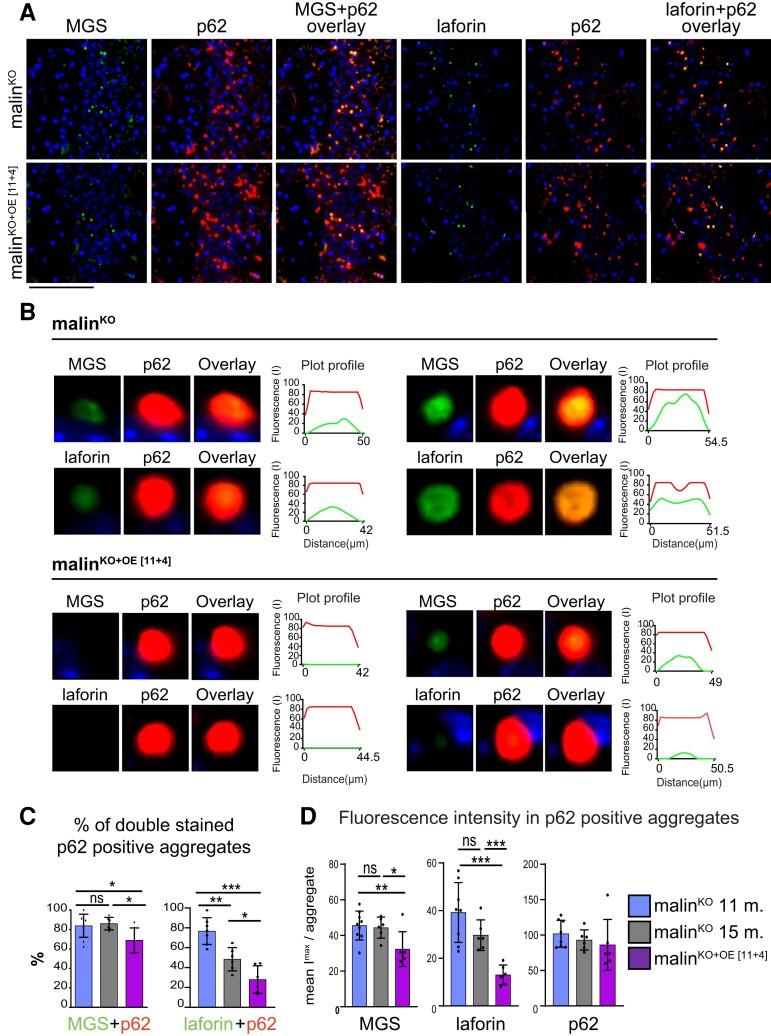
**Effect of late malin restoration on LB composition.** (**A**) Representative immunofluorescent images from 15-month-old malin^KO^ mice and malin^KO+OE[11+4]^ mice. Co-stainings: MGS (green)/p62 (red) and laforin (green)/p62 (red), both combined with DAPI staining (blue), each channel is shown separately and as merge. Scale bar: 100 µm. (**B**) Two representative individual LBs and their immunofluorescent profile along a line crossing the geometrical centre are shown for each group. The profiles correspond to MGS (green)/p62 (red) or laforin (green)/p62 (red) combinations. Each channel and merge images are shown. The size of the p62-positive particle is indicated in each graphic. (**C**) Quantification of the percentage of double-positive MGS/p62 and laforin/p62 aggregates in each group. ANOVA with Tukey's multiple comparison test and unpaired *t*-test. MGS + p62: malin^KO^ 11 months versus malin^KO^ 15 months: *P* = 0.68, malin^KO^ 11 months versus malin^KO+OE[11+4]^: *P* = 0.037, malin^KO^ 15 months versus malin^KO+OE[11+4]^: 0.0146. Laforin + p62: malin^KO^ 11 months versus malin^KO^ 15 months: *P* = 0.002, malin^KO^ 11 months versus malin^KO+OE[11+4]^: *P* ≤ 0.0001, malin^KO^ 15 months versus malin^KO+OE[11+4]^: *P* = 0.02. (**D**) Fluorescent intensity of MGS and laforin in p62-positive aggregates. ANOVA with Tukey’s multiple comparison test and unpaired *t*-test. MGS mean *I*_max_: malin^KO^ 11 months versus malin^KO^ 15 months: *P* = 0.76, malin^KO^ 11 months versus malin^KO+OE[11+4]^: *P* = 0.0135, malin^KO^ 15 months versus malin^KO+OE[11+4]^: 0.0273. Laforin mean *I*_max_: malin^KO^ 11 months versus malin^KO^ 15 months: *P* = 0.1178, malin^KO^ 11 months versus malin^KO+OE[11+4]^: *P* = 0.0004, malin^KO^ 15 months versus malin^KO+OE[11+4]^: 0.0003. p62 mean *I*_max_: malin^KO^ 11 months versus malin^KO^ 15 months: *P* = 0.37, malin^KO^ 11 months versus malin^KO+OE[11+4]^: *P* = 0.311, malin^KO^ 15 months versus malin^KO+OE[11+4]^: 0.67. *P-value ≤ 0.05*(*), *P-value < 0.01* (**), and *P-value < 0.001* (***). In all graphics, the data are shown as mean ± SD. Each dot represents one mouse.

Finally, we analysed the composition of individual aggregates after malin expression. To this end, we co-stained brain slices with anti-MGS/anti-p62 or anti-laforin/anti-p62 combination of antibodies. In the vast majority of LBs in malin^KO^ mice, p62 colocalized with MGS and p62. In contrast, malin^KO+OE[11+4]^ mice showed a considerable reduction in the number of laforin/p62- and MGS/p62-positive aggregates (Fig. 6A). To visualize the reduction in laforin or MGS in the p62 aggregates in malin^KO+OE[11+4]^ animals, we generated a plot profile of representative p62-positive neuronal LBs from malin^KO^ and malin^KO+OE[11+4]^ mice (Fig. 6B). We also measured the percentage of aggregates that were positive for MGS and p62, or for laforin and p62. These percentages were significantly reduced in mice with restored malin expression (Fig. 6C). Moreover, representation of the fluorescence intensity of laforin and MGS quantified at each p62-positive aggregate again revealed that malin expression decreased the laforin and MGS content of these aggregates (Fig. 6D).

### Malin expression ameliorates neuroinflammation at an advanced stage of Lafora disease

**Figure 7:**
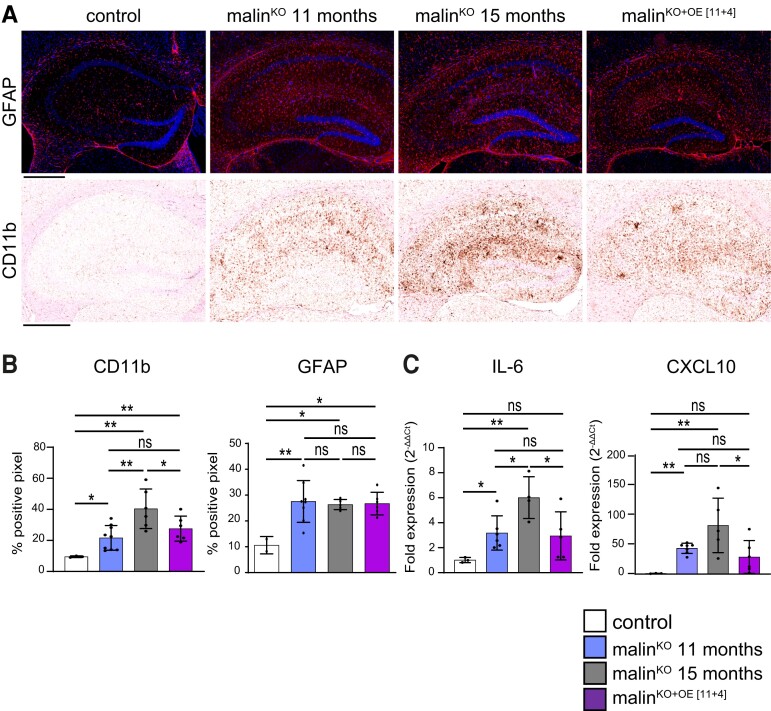
**Inflammatory response after malin restoration at advanced stage of Lafora disease.** (**A**) Astrogliosis and microgliosis were determined by immunostaining using anti-GFAP (red, combined with DAPI, blue) or anti-CD11b antibodies, respectively. Representative images from the hippocampus are shown. Scale bar: 500 µm. (**B**) Quantification of GFAP and CD11b stainings represented as percentage of positive pixel in the hippocampal region. *N = 3* sections per mice, *3*–*6 mice*. (**C**) RNA from total brain homogenates was analysed for quantification of the expression of pro-inflammatory genes by qPCR: *IL-6*, *CXCL10*. Graphics show the relative expression levels (2^−ΔΔCt^). Unpaired *t*-test. *P-value ≤ 0.05*(*), *P-value < 0.01* (**), and *P-value < 0.001* (***). CD11b: control versus malin^KO[11+4]^ 11 months: *P* = 0.028, control versus malin^KO^ 15 months: *P* = 0.0049, control versus malin^KO+OE[11+4]^: *P* = 0.0073, malin^KO^ 11 months versus malin^KO^ 15 months: *P* = 0.0037, malin^KO^ 11 months versus malin^KO+OE[11+4]^: *P* = 0.18, malin^KO^ 15 months versus malin^KO+OE[11+4]^: *P* = 0.064. GFAP: control versus malin^KO^ 11 months: *P* = 0.002, control versus malin^KO^ 15 months: *P* = 0.005, control versus malin^KO+OE[11+4]^: *P* = 0.035, malin^KO^ 11 months versus malin^KO^ 15 months: *P* = 0.75, malin^KO^ 11 months versus malin^KO+OE[11+4]^: *P* = 0.82, malin^KO^ 15 months versus malin^KO+OE[11+4]^: *P* = 0.86. IL6: control versus malin^KO^ 11 months: *P* = 0.03, control versus malin^KO^ 15 months: *P* = 0.004, control versus malin^KO+OE[11+4]^: *P* = 0.14, malin^KO^ 11months versus malin^KO^ 15 months: *P* = 0.018, malin^KO^ 11 months versus malin^KO+OE[11+4]^: *P* = 0.8, malin^KO^ 15 months versus malin^KO+OE[11+4]^: *P* = 0.018. CXCL10: control versus malin^KO^ 11 months: *P* = 0.001, control versus malin^KO^ 15 months: *P* = 0.0025, control versus malin^KO+OE[11+4]^: *P* = 0.1156, malin^KO^ 11months versus malin^KO^ 15 months: *P* = 0.0794, malin^KO^ 11 months versus malin^KO+OE[11+4]^: *P* = 0.8, malin^KO^ 15 months versus malin^KO+OE[11+4]^: *P* = 0.047. Each dot represents one mouse (*n = 3*–*6 mice*). Data are shown as mean ± SD.

Inflammation is a key pathological trait of Lafora disease.^26^ The progression of microglial and astrocytic activation during the course of the disease in malin^KO^ mice has been described previously.^11^ We examined these inflammatory cell populations in malin^KO+OE[11+4]^ mice by CD11b and GFAP immunostaining, respectively. CD11b-positive microglia were significantly increased from 11- to 15-month-old malin^KO^ mice. Importantly, malin^KO+OE[11+4]^ mice showed clearly lower levels of CD11b staining than age-matched malin^KO^ animals, and comparable levels to those found in 11-month-old malin^KO^ mice (Fig. 7A and B). In contrast, GFAP staining was not increased in malin^KO^ mice from 11 to 15 months of age. Therefore, undistinguishable GFAP levels were found among 11-, 15- month-old malin^KO^ and malin^KO+OE[11+4]^ mice (Fig. 7A and B). It has been described that the expression of pro-inflammatory genes such as *IL-6* and *CXCL10* is increased along the course of the disease, associated to the activation of astrocytes and microglia.^11,14,27^ In line with the previous results, the restoration of malin expression reduced the levels of inflammatory genes compared with age-matched malin^KO^ mice (Fig. 7C). Taken together, these results indicate that malin restoration causes a significant amelioration of the inflammatory response.

### Effects of malin expression on the skeletal muscle

**Figure 8:**
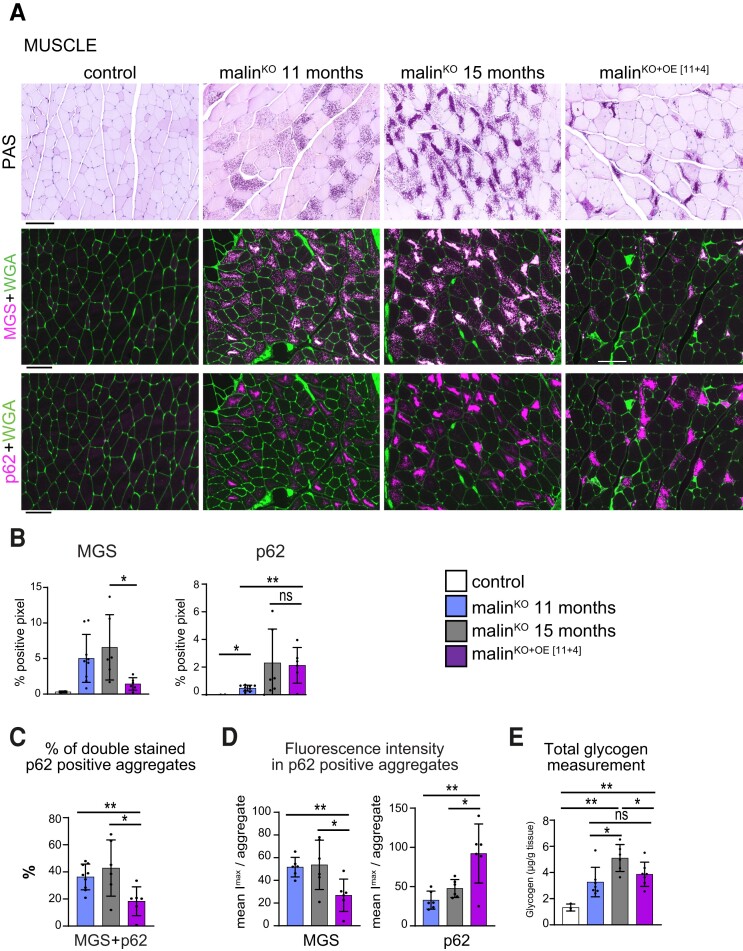
**Effect of late malin expression on skeletal muscle.** (**A**) PAS staining and immunofluorescent images from quadriceps muscles are shown for each group. Co-stainings of MGS/p62 were performed. For simplicity, MGS and p62 are shown independently. MGS (magenta, middle panels) and p62 (magenta, lower panels) were combined with agglutinin (WGA) to visualize the fibres (green). Scale bar: 100 µm. (**B**) The amount of MGS and p62 in the tissue was quantified as percentage of positive pixel in each group and represented as mean ± SD. Unpaired *t*-test, MGS % positive pixel: malin^KO^ 15 months versus malin^KO+OE[11+4]^: *P* = 0.0022; p62% positive pixel: malin^KO^ 15 months versus malin^KO+OE[11+4]^: *P* = 0.88. *P-value ≤ 0.05*(*), *P-value < 0.01* (**), and *P-value < 0.001* (***). Unpaired *t*-test, *P-value ≤ 0.05*(*), *P-value < 0.01* (**), and *P-value < 0.001* (***). (**C**) Quantification of the percentage of double-positive MGS/p62 individual aggregates. ANOVA with Tukey’s multiple comparison test and unpaired *t*-test; malin^KO^ 11 months versus malin^KO^ 15 months: *P* = 0.41, malin^KO^ 11months versus malin^KO+OE[11+4]^: *P* = 0.0045, malin^KO^ 15 months versus malin^KO+OE[11+4]^: *P* = 0.0277. (**D**) Representation of the MGS fluorescence intensity in p62-positive individual aggregates in each group. ANOVA with Tukey’s multiple comparison test and unpaired *t*-test. MGS: malin^KO^ 11 months versus malin^KO^ 15 months: *P* = 0.83, malin^KO^ 11months versus malin^KO+OE[11+4]^: *P* = 0.0045, malin^KO^ 15 months versus malin^KO+OE[11+4]^: *P* = 0.03. p62: malin^KO^ 11 months versus malin^KO^ 15 months: *P* = 0.048, malin^KO^ 11 months versus malin^KO+OE[11+4]^: *P* = 0.0041, malin^KO^ 15 months versus malin^KO+OE[11+4]^: *P* = 0.019. (**E**) Total amount of glycogen in muscle was determined (µg/g of tissue) in each group. Data are shown as mean ± SD, each dot represents a mouse. ANOVA with Tukey's multiple comparison test and unpaired *t*-test: control versus malin^KO^ 11 months: *P* = 0.017, control versus malin^KO^ 15 months: *P* = 0.0002, control versus malin^KO+OE[11+4]^: *P* = 0.0012, malin^KO^ 11months versus malin^KO^ 15 months: *P* = 0.011, malin^KO^ 11 months versus malin^KO+OE[11+4]^: *P* = 0.3, malin^KO^ 15 months versus malin^KO+OE[11+4]^: *P* = 0.046. *P-value < 0.01* (**), and *P-value < 0.001* (***). Unpaired *t*-test, *P-value ≤ 0.05*(*), *P-value < 0.01* (**), and *P-value < 0.001* (***). *n = 3*–*6 mice*.

In mouse models of Lafora disease and patients with this condition, LBs accumulate not only in the brain but also in skeletal muscles.^11,23,28^ Therefore, we also studied the effect of malin restoration on the quadriceps muscle of malin^KO+OE[11+4]^ mice. PAS staining revealed the presence of LBs in malin^KO+OE^ mice, although they consisted of fewer, larger aggregates instead of the spread small ones characteristic of the skeletal muscles of malin^KO^ mice (Fig. 8A). The presence of MGS-positive aggregates was considerably reduced in the skeletal muscles of malin^KO+OE[11+4]^ compared with age-matched malin^KO^ mice (Fig. 8A and B). In contrast, the quantification of p62-positive pixels revealed no differences between malin^KO+OE[11+4]^ and 15-month-old malin^KO^ mice. Similar to the results obtained in the brain, malin expression reduced the percentage of p62-positive aggregates that also contained MGS (Fig. 7C). Quantifications of the MGS fluorescence intensity of p62-positive aggregates also showed that malin restoration reduced the MGS content of the aggregates (Fig. 7D). Interestingly, we detected an overall increase in p62 in malin^KO+OE[11+4]^ muscles. Finally, total glycogen level was decreased in malin^KO+OE[11+4]^ compared with age-matched malin^KO^ mice (Fig. 8E), and comparable to that of 11-month-old malin^KO^ animals. These observations thus indicate that the restoration of malin expression brought about an overall arrest in glycogen accumulation.

In all, these results indicate that malin restoration at an advanced stage of the disease halts the accumulation of new LBs also in muscle and that it is able to degrade LB-bound MGS but not p62.

## Discussion

Gene therapy is an emerging therapeutic tool for neurodegenerative diseases.^29,30^ In recent years, the molecular tools needed for gene replacement have been improved for use in the brain.^30^ As a monogenic disorder, Lafora disease is a good candidate for a gene therapy-based strategy. Here we sought to assess the effect of malin restoration on the course of the disease in the context of malin deficiency, which would constitute a proof of principle for a gene replacement therapy for Lafora disease.

The mechanism by which malin and laforin prevent the accumulation of LBs is not known. These proteins could act preventing the formation of insoluble glycogen and/or promoting its degradation once formed. We induced the expression of malin at early and advanced stages of the disease (4- and 11-month-old malin^KO^ mice, respectively). Malin did not promote the removal of pre-existing LBs, as shown by PAS staining and total glycogen measurements. This observation indicates that malin is not capable of inducing the degradation of the insoluble glycogen that makes up the core of LBs. However, our results indicate that malin arrested LB accumulation in the brain and skeletal muscle even when its expression was induced at an advanced stage of the disease.

Interestingly, malin induced the degradation of MGS and laforin bound to LBs, thereby confirming previous results obtained *in vitro*.^2^ This finding indicates that the restored malin interacts with laforin, which is bound to glycogen in the LBs,^7^ and promotes the degradation of proteins present in the LBs such as MGS.^6^ Strikingly, p62 levels remained high after malin restoration. This finding suggests that the abnormal glycogen that forms the core of the LBs remains in the tissue bound together by p62, and probably other protein components. In this regard, we have recently described that p62-driven aggregation of abnormal glycogen reduces its toxicity.^13^

One of the most important aspects of the progression of the disease is the appearance of neuroinflammation, which occurs through the activation of astrocytes and microglia. In recent years, this response has been gaining relevance and it has been proposed as a target for therapeutic strategies for Lafora disease.^26,31^ Although the mechanism of microglial activation in Lafora disease is still unknown, it involves the accumulation of glycogen in the form of LBs in astrocytes, as we have recently demonstrated.^14^ Herein we show that the restoration of malin expression, even at an advanced stage of the disease, ameliorates the inflammatory response, as seen by the reduction in the activation of microglia and inflammatory genes. This effect on microglial response might be the result of blocking of the formation of new LBs but also of the changes in the protein composition of the remaining glycogen aggregates. The positive impact of malin on the microglial inflammatory response is of particular interest since it proves that it is advantageous compared with strategies based on blocking MGS expression, which are not able to reduce astrogliosis, microgliosis, or other inflammatory markers when applied at advanced stages.^11^

Given that malin is a mono-exonic gene and therefore has a small gene locus span, a malin gene replacement approach offers the possibility of including the native promoter and regulatory sequences in the appropriate vector, thereby strengthening the safety of the approach. Future work is required to evaluate the potential of laforin restoration oriented to treat laforin-deficient patients.

In conclusion, malin restoration represents a promising therapeutic strategy for malin-deficient patients as it is capable of reducing the accumulation of LBs, degrading LB components, and ameliorating inflammation. This is the first time that a therapeutic strategy for Lafora disease has shown effectiveness at advanced stages. Our results prompt further research into molecular tools for malin expression to be used as a gene replacement therapy-based approach.

## Supplementary Material

fcac168_Supplementary_Data

## Abbreviations

CAG =: CMV early enhancer/chicken β-actin
CAL =: corpora amylacea like
ERT2 =: oestrogen receptor ligand binding domain 2
LB/LBs =: Lafora body/Lafora bodies
MGS =: muscle glycogen synthase
nLBs =: neuronal Lafora bodies
PAS =: periodic acid-Schiff
