# Supplementary material for: Malin restoration as proof of concept for gene therapy for Lafora disease
Source: Brain Commun. 2022 Jun 23;4(4):fcac168. doi: 10.1093/braincomms/fcac168 (PMC9260307; doi:10.1093/braincomms/fcac168)
Supplement: fcac168_Supplementary_Data [file fcac168_supplementary_data.pdf]

Supplementary material

Supplementary Figure 1.

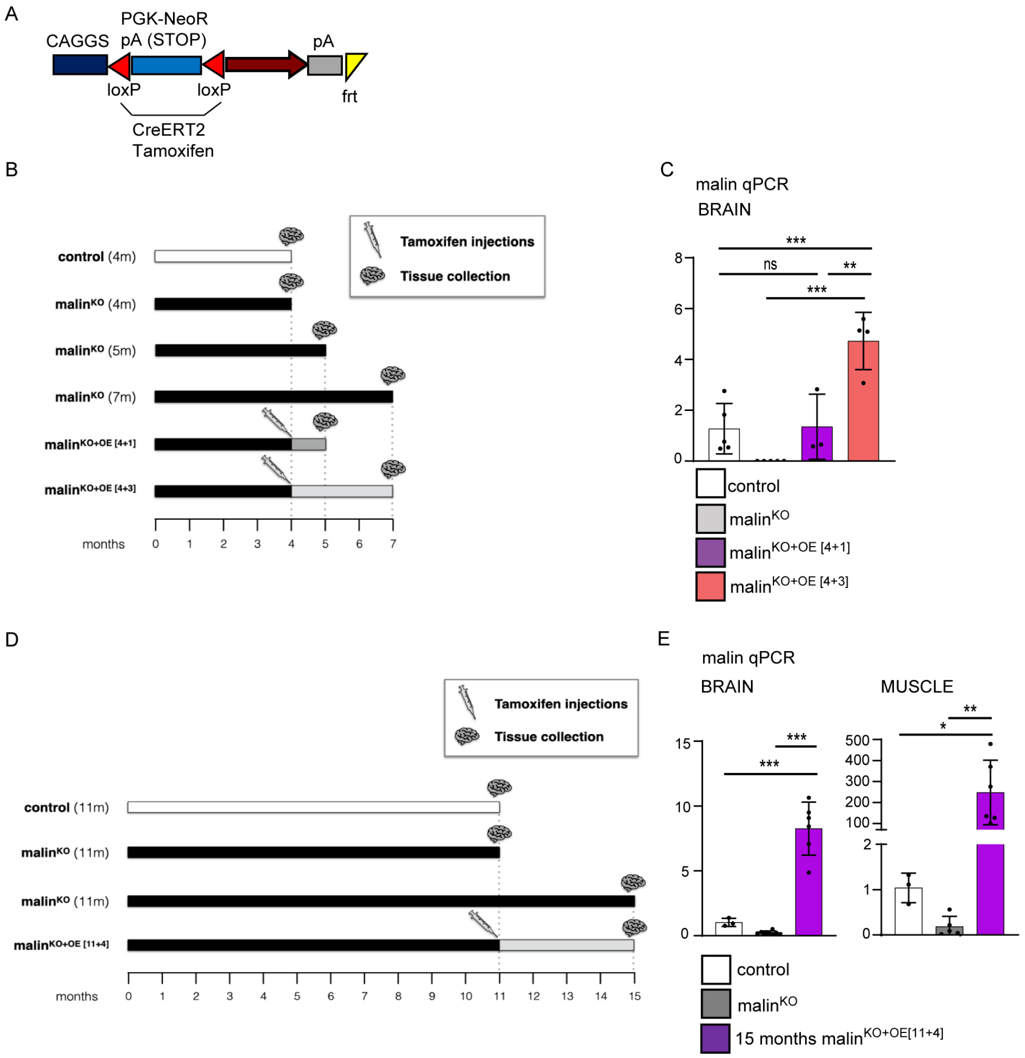

Supplementary Figure 1. Malin mRNA expression levels in brain and skeletal muscle. (**A**) Generation of the malin^{KO+OE} mice colony: conditional malin expression vector containing a floxed stop codon following the ubiquitous promoter CAG. (B) Schematic design of the experiment: malin restoration at 4 months and analysis after 1 or 3 months of malin expression. (C) Malin mRNA expression level in brain samples from 4- and 11-month-old control, malin$^{KO+OE[4+1]}$, malin$^{KO+OE[4+3]}$ and malin$^{KO+OE[11+4]}$ mice. ANOVA with Tukey´s multiple comparison test. Control versus malin$^{KO+OE[4+1]}$:p=0.92, Control versus malin$^{KO+OE[4+3]}$:p=0.001, malin^{KO} versus malin$^{KO+OE[4+3]}$:p=0.0002, malin$^{KO+OE[4+1]}$ versus malin$^{KO+OE[4+3]}$:p=0.01. (D) Schematic design of the experiment: malin restoration at 11-

month-old malin^{KO} mice and analysis after 4 months of malin expression. (E) Malin mRNA expression level in brain and quadriceps muscle from control and malin$^{KO+OE[11+4]}$ mice. Measured by qPCR from total tissue homogenate and expressed as relative expression ($2^{-\Delta\Delta Ct}$) compared to control wt mice. ANOVA with Tukey´s multiple comparison test. Brain: control versus malin$^{KO+OE[11+4]}$: p=0.0006, malin^{KO} versus malin$^{KO+OE[11+4]}$: p=0.0001. Muscle: control versus malin$^{KO+OE[11+4]}$: p=0.03, malin^{KO} versus malin$^{KO+OE[11+4]}$: p=0.008. *p value* $\leq$ *0.01* (**), and *p value* $\leq$ *0.001* (***). Unpaired t-test, *p value*$\leq$*0.05*(*), *p value* $\leq$ *0.01* (**), and *p value* $\leq$ *0.001* (***). Each dot represents one mouse.

Supplementary Figure 2.

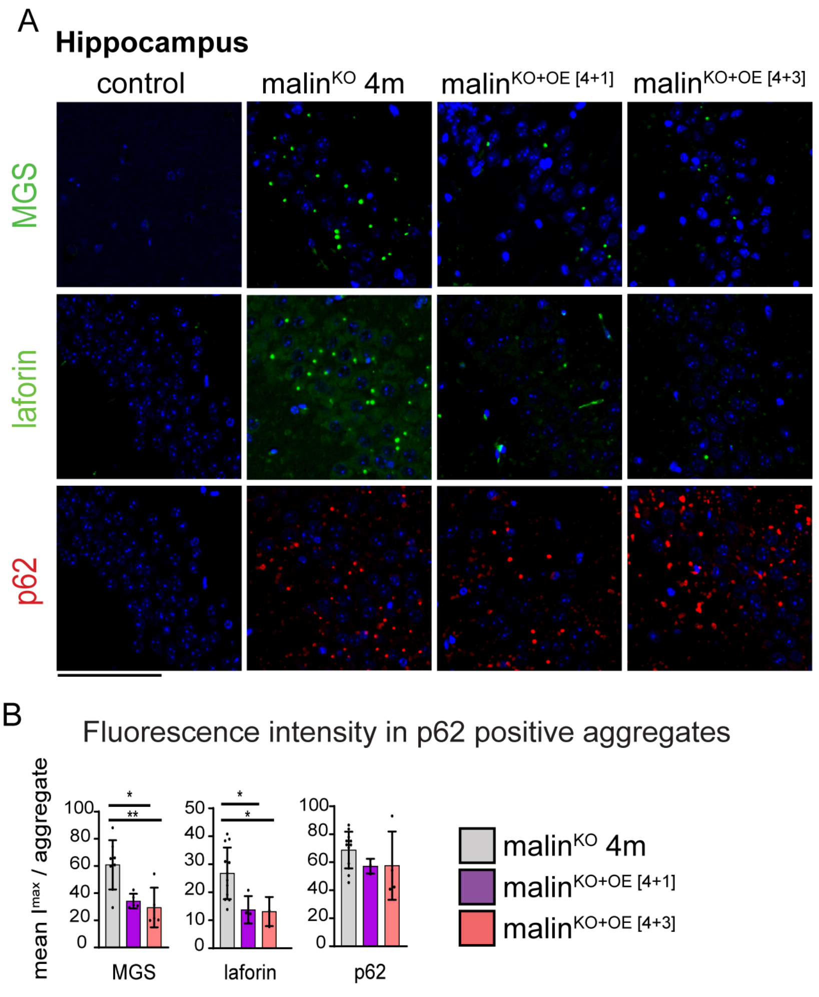

Supplementary Figure 2. LB component analysis after malin restoration of young malin^{KO} mice. (A) Representative immunofluorescence images from the CA2/CA3

hippocampal region obtained with anti- MGS (green), anti-laforin (green) or anti-p62 (red) stainings in combination with DAPI (blue). 4-month-old control and malin^{KO}, malin$^{KO+OE[4+1]}$ and malin$^{KO+OE[4+3]}$ mice are shown. Scale bar: 100 μm. (B) Fluorescent intensity of MGS and laforin in p62-positive LBs from 4-month-old malin^{KO}, malin$^{KO+OE[4+1]}$ and malin$^{KO+OE[4+3]}$ mice. Data are shown as mean±SD. ANOVA with Tukey´s multiple comparison test and unpaired t-test. MGS: malin^{KO} 4 months versus malin$^{KO+OE[4+1]}$: p=0.09, malin^{KO} 4 months versus malin$^{KO+OE[4+3]}$: p=0.0077, malin$^{KO+OE[4+1]}$ versus malin$^{KO+OE[4+3]}$: p=0.52. Laforin: malin^{KO} 4 months versus malin$^{KO+OE[4+1]}$: p=0.019, malin^{KO} 4 months versus malin$^{KO+OE[4+3]}$: p=0.03, malin$^{KO+OE[4+1]}$ versus malin$^{KO+OE[4+3]}$: p=0.87. p62: malin^{KO} 4 months versus malin$^{KO+OE[4+1]}$: p=0.16, malin^{KO} 4 months versus malin$^{KO+OE[4+3]}$: p=0.25, malin$^{KO+OE[4+1]}$ versus malin$^{KO+OE[4+3]}$: p=0.97. Each dot represents one mouse (*n=4-8* as indicated).

Supplementary Figure 3.

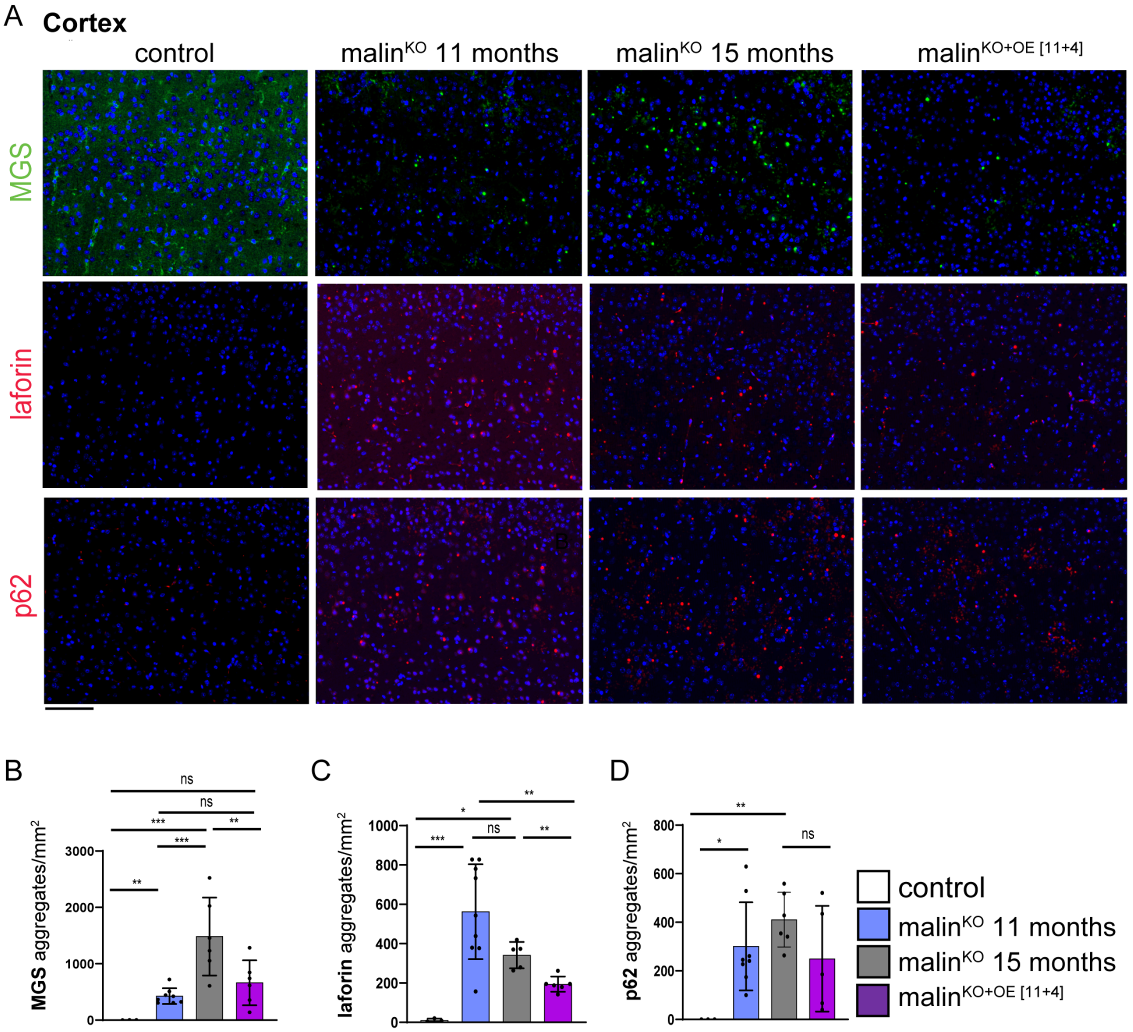

Supplementary Figure 3. MGS, laforin and p62 analysis in the cortex of malin^{KO} mice after malin restoration at advanced stage of Lafora disease. (A) Immunofluorescence images from prefrontal cortical region using anti-MGS (green), anti-laforin (red) or anti-p62 (red) antibodies combined with DAPI staining in each group. (B) Quantification of number of particles per area (mm^2) in the cortex stained with anti-MGS, anti-laforin and anti-p62 antibodies. (C) Scale bar: 100 µm. In all graphics, data are shown as mean±SD, each dot represents one mouse, *n=3-6* as indicated in the graphic. ANOVA with Tukey´s multiple comparison test and unpaired t-test. MGS: control versus malin^{KO} 11 months: p=0.007, control versus malin^{KO} 15 months: p=0.089, control versus malin$^{KO+OE[11+4]}$: p=0.02, malin^{KO} 11months versus malin^{KO} 15 months: p=0.0011, malin^{KO} 11 months versus

malin$^{KO+OE[11+4]}$: p=0.35, malin^{KO} 15 months versus malin$^{KO+OE[11+4]}$: p=0.00196. Laforin: control versus malin^{KO} 15 months: p=0.04, malin^{KO} 11months versus malin^{KO} 15 months: p=0.07, malin^{KO} 11 months versus malin$^{KO+OE[11+4]}$: p=0.0028, malin^{KO} 15 months versus malin$^{KO+OE[11+4]}$: p=0.0013. p62: control versus malin^{KO} 11 months: p=0.02, control versus malin^{KO} 15 months: p=0.0005, malin^{KO} 11months versus malin^{KO} 15 months: p=0.2, malin^{KO} 11 months versus malin$^{KO+OE[11+4]}$: p=0.65, malin^{KO} 15 months versus malin$^{KO+OE[11+4]}$: p=0.147. *p value≤0.05*(*), *p value ≤ 0.01* (**), and *p value ≤ 0.001* (***). Unpaired t-test, *p value≤0.05*(*), *p value ≤ 0.01* (**), and *p value ≤ 0.001* (***).

Supplementary Figure 4.

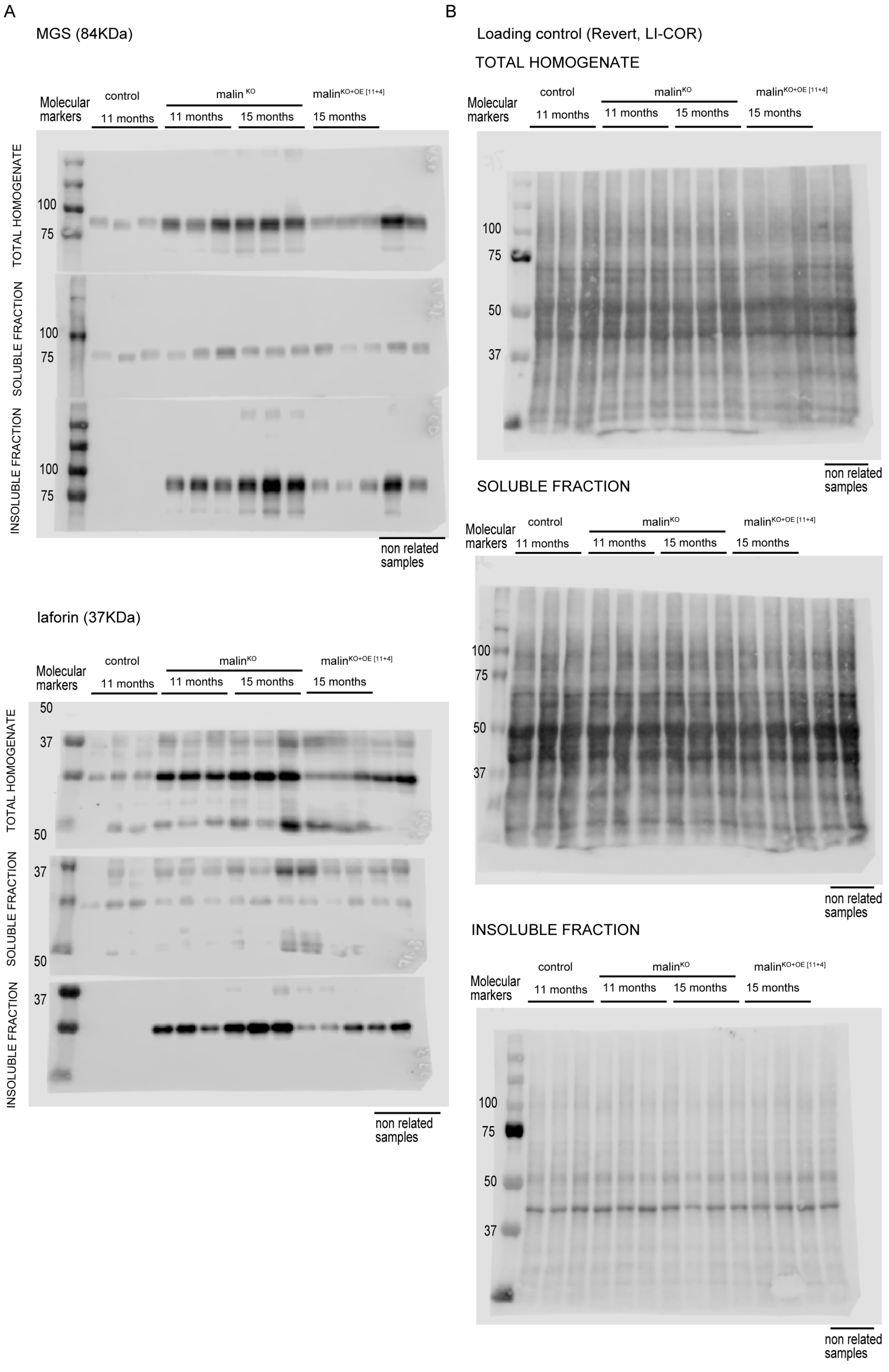

Supplementary Figure 4. Full western blot images corresponding to cropped blots shown in Figure 4. Each western blot contains triplicates for each group. All the samples from each group were run in parallel. (A) To incubate the membranes with two antibodies, they were cut above the 50KDa molecular marker, MGS was incubated in the upper part, laforin in the lower part. B) For each western blot, quantification was done with the loading control signal from the same blot (Revert signal, LICOR). The antibodies had been tested and used previously.

Supplementary Figure 5

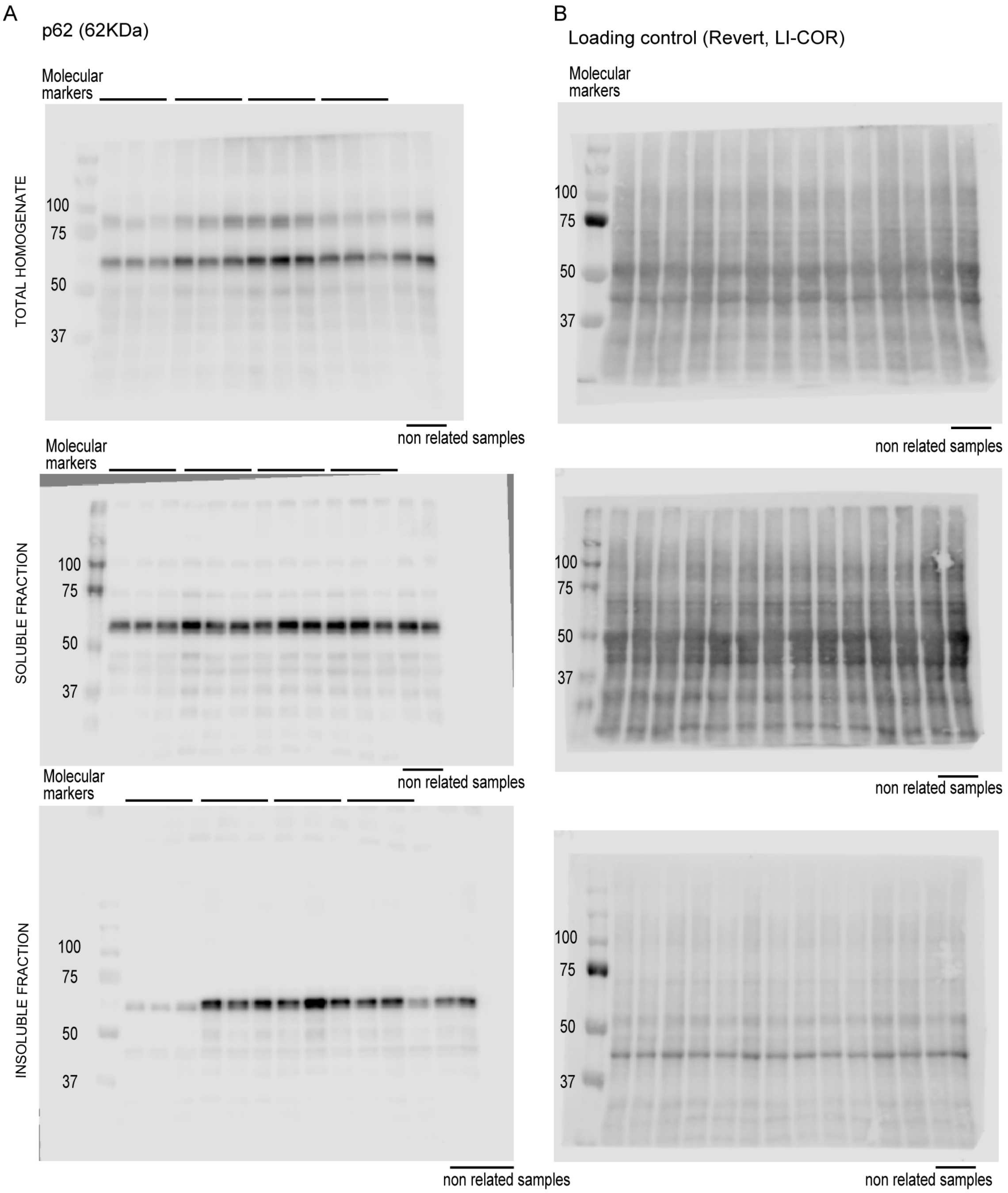

Supplementary Figure 5. Full western blot images corresponding to cropped blots shown in Figure 4. (A) Full images of the western blots shown for p62. Each western blot contains triplicates for each group. Gels for p62 were prepared in the same manner and with every sample tested for MGS and laforin. Molecular markers are indicated in the figure. (B) Full images corresponding to the loading control (Revert signal, LICOR). The antibody had been tested and used previously.
